# Deacetylation of Glutaminase by HDAC4 contributes to Lung Cancer Tumorigenesis

**DOI:** 10.7150/ijbs.69882

**Published:** 2022-07-04

**Authors:** Tao Wang, Zhuo Lu, Tianyu Han, Yanan Wang, Mingxi Gan, Jian-Bin Wang

**Affiliations:** 1 School of Basic Medical Sciences, Nanchang University, Nanchang, 330031, P. R. China.; 2 School of Life Sciences, Nanchang University, Nanchang, 330031, P.R. China.; 3 Jiangxi Institute of Respiratory Disease, The First Affiliated Hospital of Nanchang University, Nanchang, 330006, P.R. China.

**Keywords:** glutaminase, acetylation, HDAC4, TRIM21, non-small cell lung cancer

## Abstract

Inhibiting cancer metabolism via glutaminase (GAC) is a promising strategy to disrupt tumor progression. However, mechanism regarding GAC acetylation remains mostly unknown. In this study, we demonstrate that lysine acetylation is a vital post-translational modification that inhibits GAC activity in non-small cell lung cancer (NSCLC). We identify that Lys311 is the key acetylation site on GAC, which is deacetylated by HDAC4, a class II deacetylase. Lys311 acetylation stimulates the interaction between GAC and TRIM21, an E3 ubiquitin ligase of the tripartite motif (TRIM) family, therefore promoting GAC K63-linked ubiquitination and inhibiting GAC activity. Furthermore, GAC^K311Q^ mutation in A549 cells decreases cell proliferation and alleviates tumor malignancy. Our findings reveal a novel mechanism of GAC regulation by acetylation and ubiquitination that participates in non-small cell lung cancer tumorigenesis.

## Introduction

Reprogramming of cellular metabolism is a hall marker of tumorigenesis. Transformed cells often converts the majority of their glucose to lactate regardless of O_2_ availability (Warburg effect), as well as increases the use of glutamine as carbon and nitrogen source 1, 2. Glutamine is the amplest amino acid in cells that is both non-essential and conditionally essential in humans. When body is under stressful conditions, cells may have a heavy glutamine requirement that surpasses its synthetic ability 3. In tumor cells, glutamine is converted to alpha-ketoglutarate to support bioenergetics through the tricarboxylic acid cycle or join a biosynthesis pathway via aminotransferase 4, 5. Aberrant glutamine metabolism has also been implicated in regulating cell signaling and epigenetics 6, 7.

Glutaminase catalyzes the first step in glutaminolysis that converts glutamine to glutamate and nitrogen 8. Kidney-type glutaminase and liver-type glutaminase are the two main isoforms of glutaminase in humans. KGA and GAC are the two different splicing variants of GSL1 9. GAC is the predominant GLS isoform in a variety of tumor cells and is demonstrated to be correlated with tumor growth 10-13. This vital function of glutaminase in glutaminolysis makes it a promising target for tumor therapy.

The regulatory mechanisms regarding regulation of GAC expression and activity have been deeply investigated. Several oncogenes (KRAS, c-Myc) and Hypoxia-inducible factor (HIF) are reported to drive the up-regulation of GAC at transcriptional level 14-16. Alternative splicing of GLS1 regulated by CCAT2, a long non-coding RNA (lncRNA), results in the expression of GAC rather than KGA 17. High-throughput mass spectrometry platform have screened out a number of post-translational modifications (PTMs) on GAC. Activation of NF-κB-PKCε signaling enhances GAC activity by promoting its phosphorylation at residue Ser314 18. Sirt5, a NAD^+^-dependent deacylase, stabilizes GAC through the desuccinylation of residue Lys164 13. Several sites of acetylated lysine are identified in GAC, some of which are located within its catalytic domain, though the functions and mechanisms of acetylation on GAC remain largely unclear 19-22. Here, we identified Lys311 as a key acetylation site in regulating GAC activity and HDAC4 as the deacylase responsible for its deacetylation. We found that acetylation of Lys311 promoted GAC ubiquitination and inhibited its activity. Furthermore, we demonstrated that as a E3 ligase, TRIM21 could ubiquitinate GAC in K63-linkage following GAC acetylation. In conclusion, our study revealed a novel regulatory mechanism of GAC by acetylation and ubiquitination that participates in tumorigenesis in NSCLC.

## Materials and Methods

### Cell culture

293T cells were cultured in DMEM (Genview, GD3123) supplemented with 10% fetal bovine serum (Gibco). NSCLC cell lines (A549, H1299, H292) were cultured in RPMI 1640 (Gibco, C11875500BT) supplemented with 10% fetal bovine serum (Gibco). All cells were cultured at 37 °C containing 5% CO_2_. All cell lines are purchased from ATCC.

### Reagents

Primary antibodies against Actin (66,009-1-lg), HDAC4 (17449-1-AP), UB (10201-2-AP) and V5 (14440-1-AP) were purchased from Proteintech. K63-linkage-specific polyubiquitin antibody (920435621), K48-linkage-specific polyubiquitin antibody (920438081) and anti-TRIM21 antibody (92043) were ordered from Cell Signaling Technology. Anti-acetyl-Lysine antibody (06-933) was purchased from Millipore. Anti-GAC antibody (ab93434) were ordered from Abcam. Anti-HA mouse monoclonal antibody was purchased from Thermo Fisher Scientific (26183). The HDAC4 siRNAs (OriGene, SR306523) and TRIM21 siRNAs (OriGene, SR304594) were purchased from OriGene. The polyclonal antibody against anti-acetyl-lysine 311 GAC (GAC-AcK311) was manufactured by Shanghai Genomics Inc (antigen sequence: VHRYVGK(Ac)EPSGLR; immunogen: Peptide-KLH conjugated). In summary, antigen peptide (VHRYVGK(Ac)EPSGLR) was synthetized and conjugated with KLH. Then immunogen mixed with Freund's adjuvant was subcutaneously injected to rabbit every week for 4 times. Rabbit was sacrificed and serum was collected. Antibody against GAC-AcK311 was purified from serum using unacetylated antigen peptide (VHRYVGKEPSGLR).NAM (sigma, 72340) and TSA (sigma, V900931) were purchased from sigma.

### Gene knockdown and overexpression

Cells were seeded 12 h prior to transfection. The siRNAs transfection was performed with SuperFectin siRNA Transfection Reagent (Pufei, 2103-100). Plasmid transfection was performed with SuperFectin DNA Transfection Reagent kit (Pufei, 2102-100). All transfection experiments were performed according to instruction manual.

### Cell proliferation and migration assay

For cell growth assays of A549-GAC^WT^ and A549-GAC^K311Q^ stable cell lines, 5000 cells were seeded in 24-well plates in RPMI 1640 containing 10% fetal bovine serum. For cell growth assays following knockdown or overexpression of HDAC4, cells were transfected with indicated siRNAs or plasmids. 5000 cells were seeded in 24-well plates in RPMI 1640 containing 10% fetal bovine serum. Cells were fixed with 4% formaldehyde and stained in 0.1% crystal violet at the indicated time. Crystal violet was extracted using 10% acetic acid and cell growth rate was assayed by the absorbance at 595 nm.

For colony formation assay, 500 cells were seeded in 6-well plates in RPMI 1640 containing 10% fetal bovine serum. Cells were fixed with 4% formaldehyde after 10-15 days and stained in 0.1% crystal violet, then photographed.

For soft agar assay, cells were suspended in RPMI 1640 containing 10% fetal bovine serum and 0.3% agarose and then plated on a solidified layer of RPMI 1640 containing 10% fetal bovine serum and 0.5% agarose. Fresh medium with 10% fetal bovine serum and 0.5% agarose were added to the plates after 7 Days. Cell colonies were photographed after 14 days of growth.

For cell migration assay, cells were seeded in 6-well plate and transfected with indicated plasmids or siRNAs. When cells were up to 90% confluence, draw a straight line across the monolayer with 200 μl pipet tip and wash the wells with PBS for three times. Then cells were cultured in RPMI 1640 (Gibco, C11875500BT) supplemented with 1% fetal bovine serum (Gibco) and photographed at indicated time.

For pseudopodia formation assays, cells were seed in 24-well plate and transfected with indicated siRNAs for 48 h. Cells were fixed with 4% formaldehyde and incubated with PBS containing 0.25% Triton X-100 for an hour at room temperature. Then cells were incubated with TRITC-phalloidin (Sigma) for an hour at room temperature and washed with PBS. Finally, cells were mounted with DAPI Fluoromount-G mounting medium (SouthernBiotech, 0100-20) and photographed.

### Mitochondrial protein isolation and glutaminase activity assay

Mitochondria isolation kit from QIAGEN (37612) was used to isolate mitochondrial proteins according to instruction manual. Briefly, cells were centrifuged and suspended in 2 ml of lysis buffer and incubated on ice for 10 min. The cell lysates were centrifuged at 1000 × g for 10 min at 4 °C and the resulting pellets were resuspended in 1.5 ml disruption buffer using a blunt-ended syringe. The suspension was centrifuged at 6000 g for 20 min at 4 °C. The pellets were resuspended in 100 µL of storage buffer and stored at -80 °C for latter analysis.

The detailed procedures for endogenous glutaminase activity assay have been previously reported 18. For the glutaminase activity assay of ectopic expressed V5-tagged GAC, the indicated plasmids were transfected into H1299 cells. Cells were then lysed and immunoprecipitated with anti-V5 antibody. GAC activity assay were the same as above.

### Immunoprecipitation and western blot assay

Cells were lysed in NP40 lysis buffer and then cell lysates were centrifuged at 12,000 g, 4 °C for 20 min. 20 µL protein G agarose beads (Roche, 11243233001) were added to the supernatants for preclear. The supernatants were incubated with protein G agarose beads and indicated antibodies at 4 °C for 8 h. Then immunocomplexes were centrifuged at 4 °C for 3 min and NP-40 lysis buffer were used to wash the precipitates three times before subjected to western blot assay.

For western blot assay, proteins were separated on 10% or 12% SDS-PAGE gel where appropriate. Proteins were then transferred to PVDF membranes (Millipore, IPVH00010), which were blocked with 5% BSA (Genview, FA016). Next PVDF membranes were incubated with the indicated antibodies and washed 3 times with TBST (0.05% Tween-20, 150 mM NaCl and 20 mM Tris-HCl). Lastly, PVDF membranes were incubated with horseradish peroxidase-conjugated anti-rabbit (Thermo Fisher Scientific, 31460) or horseradish peroxidase-conjugated anti-mouse (Thermo Fisher Scientific, 31430) secondary antibodies. Western blot results were obtained by digital gel image analysis system (TANON 5500) and Pro-Light chemiluminescence detection kit (TIANGEN, PA112-01).

### *In vivo* xenograft assay

Three weeks old male BALB/C nude mice (Nanjing) were subcutaneously injected with a total number of 1 × 10^7^ cells. After a month, mice were sacrificed and tumors were dissected out. Tumor volume was calculated as previously reported 18. All mice were fed in the Specific Pathogen Free animal facility in the Institute of Life Science at Nanchang University.

#### Stable cell line construction

A549 cells were seeded in 100 mm cell culture dish and transfected with either pcDNA3.1-V5-GACWT or pcDNA3.1-V5-GACK311Q plasmid. After 48 h of transfection, required amount of G418 was added to the media. The media was changed every 48 h with G418 until colonies started forming. Pick up the colonies and transfer it to a 96-well plate. When cells start growing in number, gradually move them to bigger wells. Upon having sufficient cells, screen colonies for positivity by western blotting.

### Hematoxylin-eosin staining and immunohistochemistry

Tumors formed by the parental A549-GAC^WT^ and A549-GAC^K311Q^ stable cells were subjected to Immunohistochemical staining and Hematoxylin-eosin (H&E) staining conducted by Wuhan Servicebio Technology Co., Ltd. Antibodies against Ki67 (ab15580) and TTF1 (ab76013) were acquired from Abcam. Photos were obtained with Olympus IX71 microscope.

### Metabolomic analysis

ATP determination kit (AZZ066) was obtained from Thermo Fisher. The experiment was performed according to instruction manual. Metabolites' concentrations were determined by NMR analysis performed by Wuhan Anachro Technologies INC.

### Statistical analysis

All the data were showed as mean ± S.D. (standard deviation). Three independent replicates were performed for each experiment. Differences between groups were calculated by ANOVA or by Student's *t* test where appropriate. *P* < 0.05 were considered significant.

## Results

### Acetylation at K311 inhibits GAC activity

**Figure 1:**
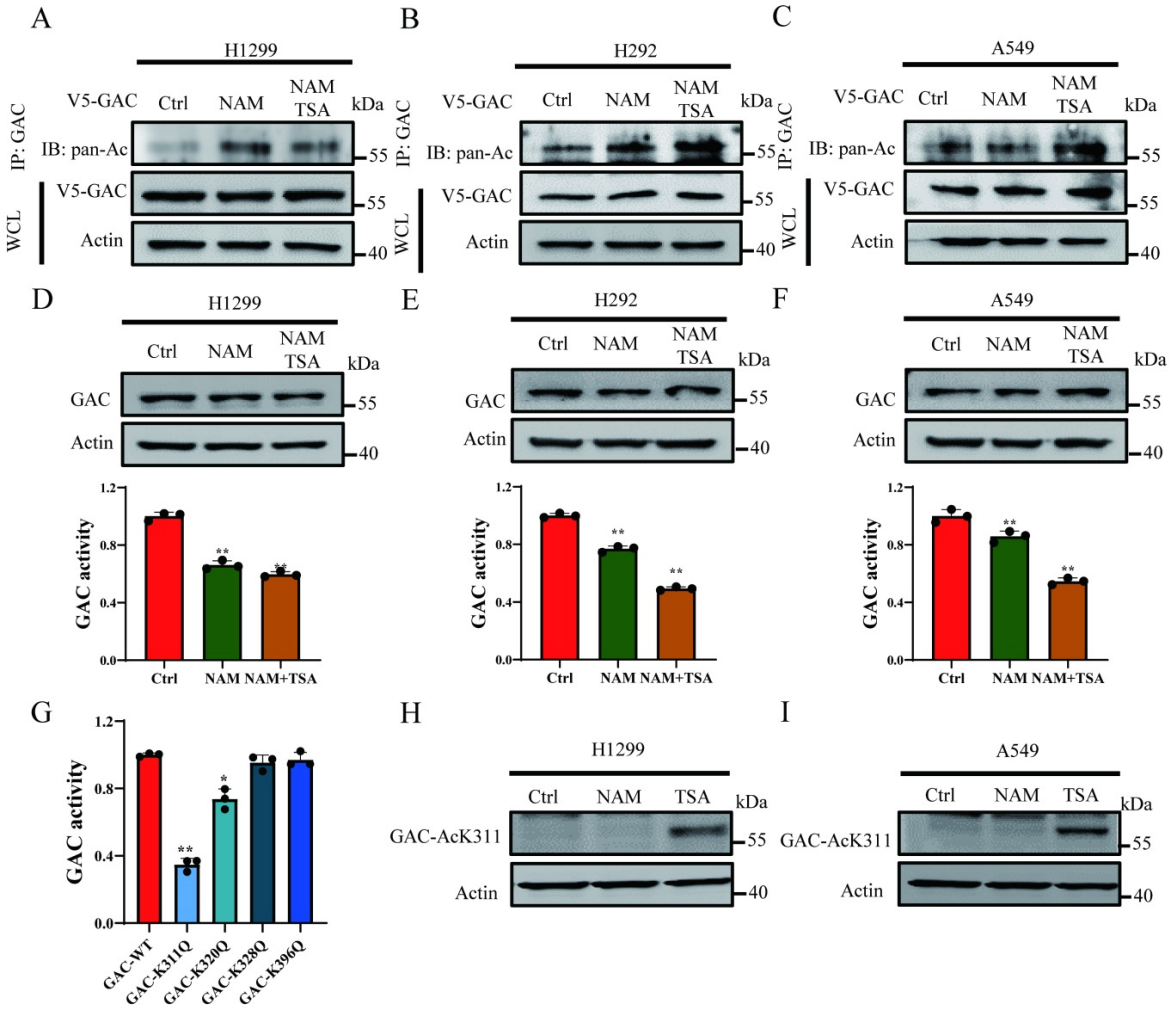
** Acetylation at K311 inhibits GAC activity. A-C** NSCLC cells (H1299, A549, H292) were transfected with V5-tagged GAC and treated with NAM and/or TSA. Western blot assay was performed. WCL: whole cell lysate. **D-F** NSCLC cells (H1299, A549, H292) were treated with NAM and/or TSA. The protein expression was determined by western blot and glutaminase activity assay was performed. **G** V5-GAC^WT^ or V5-GAC^mutants^ (K311Q, K320Q, K328Q, K396Q) plasmids were transfected into H1299 cells and glutaminase activity assay was performed. H, I H1299 **(H)** and A549 **(I)** cells were treated with NAM and TSA. GAC K311-acetylation was detected by western blot assay using anti-GAC AcK311 antibody. Data are showed as mean ± SD, n=3. *P < 0.05, **P < 0.01.

In order to prove the acetylation of GAC in NSCLC cells, V5-tagged GAC was ectopically expressed into H1299, H292 and A549 cells and immunoprecipitated with indicated antibody. Western blot results proved that GAC was strongly acetylated and trichostatin A (TSA, an inhibitor of HDAC family deacetylase) and nicotinamide (NAM, an inhibitor of SIRT family deacetylases) treatment enhanced its acetylation (Fig. 1A-C). To investigate the function of acetylation on GAC activity and stability, cells were treated with TSA and NAM and then GAC expression and activity were assayed. Our results showed that the activity of GAC decreased significantly followed with NAM and TSA treatment while its protein expression remained unchanged (Fig. 1D-F).

High-throughput mass spectrometry-based screens have identified several acetylated lysine residues on GAC. We focused on the acetylation sites which were located within catalytic domain of GAC, namely Lys311, Lys320, Lys328, Lys396, and mutated each of these residues into acetyl-mimetic glutamine. We transfected H1299 cells with wild-type GAC or GAC mutants and GAC activity was measured. We found that mutation of Lys311 significantly reduced the activity of GAC while mutation of Lys320 showed mild inhibition on GAC activity (Fig. 1G). Then antibody specific to acetylated Lys311 were manufactured to determine if Lys311 was acetylated *in vivo*. Western blot result showed a strong Lys311-acetylation signal of wild-type GAC, but not GAC^K311Q^ mutant (Fig. S1). Furthermore, Lys311-acetylated GAC was increased by TSA treatment but not NAM (Fig. 1H and I), demonstrating endogenous acetylation of GAC at Lys311 in cultured cells.

### HDAC4 is responsible for the deacetylation of GAC at K311

**Figure 2:**
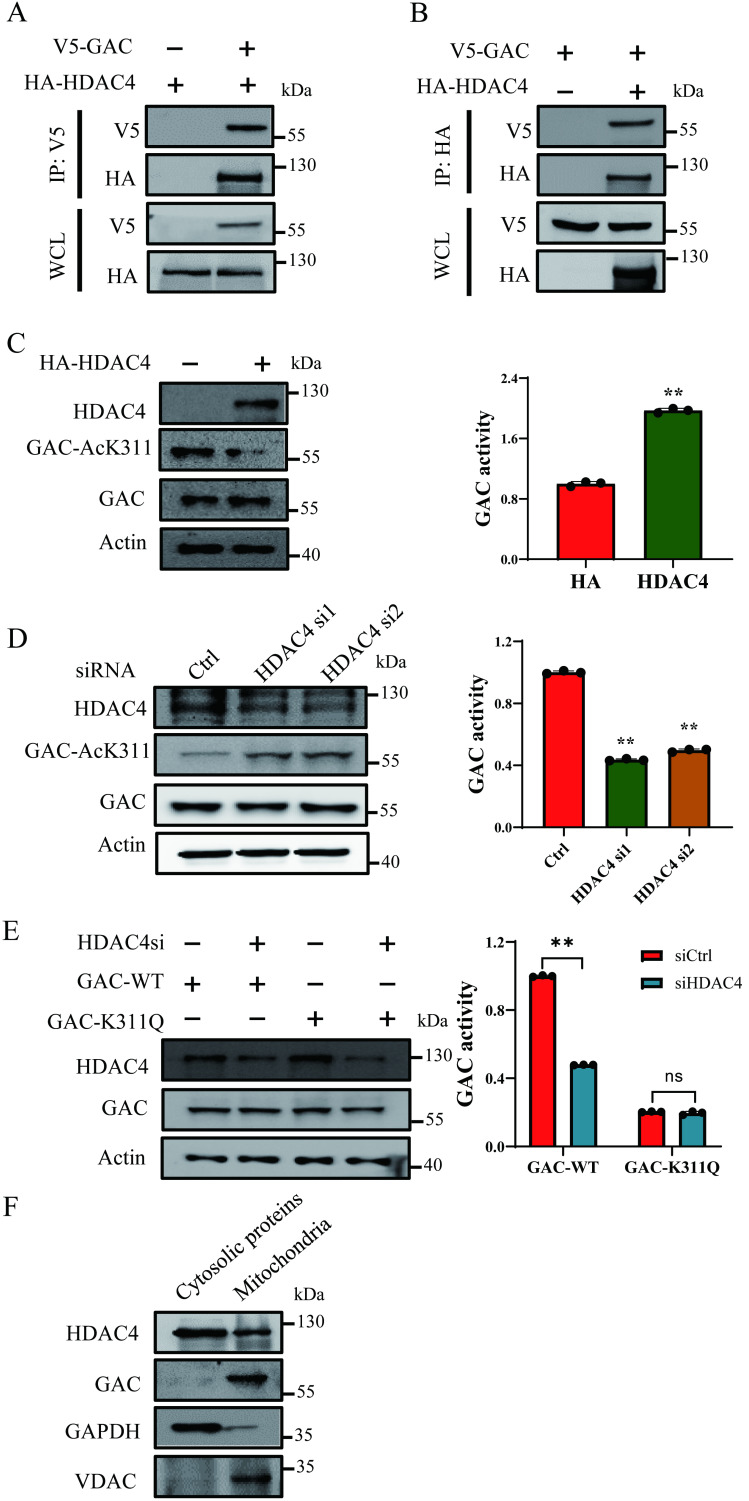
** HDAC4 is responsible for deacetylation of GAC at K311. A, B** Indicated plasmids were transfected into H1299 cells. Interaction between HDAC4 and GAC was detected by immunoprecipitation and western blot. WCL: whole cell lysate. **C** Indicated plasmids were transfected into H1299 cells. The protein expression was determined by western blot and glutaminase activity assay was performed. **D** Indicated siRNAs were transfected into H1299 cells. The protein expression was determined by western blot and glutaminase activity assay was performed. **E** Indicated plasmids and siRNAs were transfected into H1299 cells and glutaminase activity assay was performed. **F** The mitochondrial and cytosolic proteins in H1299 cells were separated and the location of HDAC4 and GAC was determined by western blot. VDAC was used as a marker of mitochondrial proteins and GAPDH was used as a marker of cytosolic proteins. Data are showed as mean ± SD, n=3. **P < 0.01, ns *P* >0.05.

TSA treatment led to increased GAC-AcK311 level while NAM treatment caused negligible GAC-AcK311 level change, suggesting that GAC K311 was deacetylated by a class I or II deacetylase. Immunoprecipitation and western blot results showed that GAC interacted strongly with HDAC4, but not with HDAC6, HDAC7 or HDAC10 (Fig. 2A, B; Fig. S2A-C). To explored the role of HDAC4 in GAC deacetylation, HDAC4 was overexpressed in H1299 and A549 cells, and the level of K311 acetylated GAC decreased significantly while GAC activity was increased (Fig. 2C; Fig. S2D). Knocking down HDAC4 increased K311 acetylation and reduced GAC activity in H1299 and A549 cells (Fig. 2D; Fig. S2E). Together, these data showed that HDAC4 deacetylated and activated GAC. Furthermore, HDAC4 knockdown in H1299 and A549 cells had little effect on the activity of GAC^K311Q^ mutant, suggesting that HDAC4 activated GAC mostly via deacetylating K311 (Fig. 2E; Fig. S2F). We then separated mitochondria from cytoplasm using mitochondrial separation kit and found that HDAC4 localized in both cytoplasm and mitochondria while GAC localized in mitochondria (Fig. 2F), suggesting that the deacetylation of GAC by HDAC4 took place in mitochondria.

### HDAC4 promoted cell proliferation and migration in NSCLC cells

**Figure 3:**
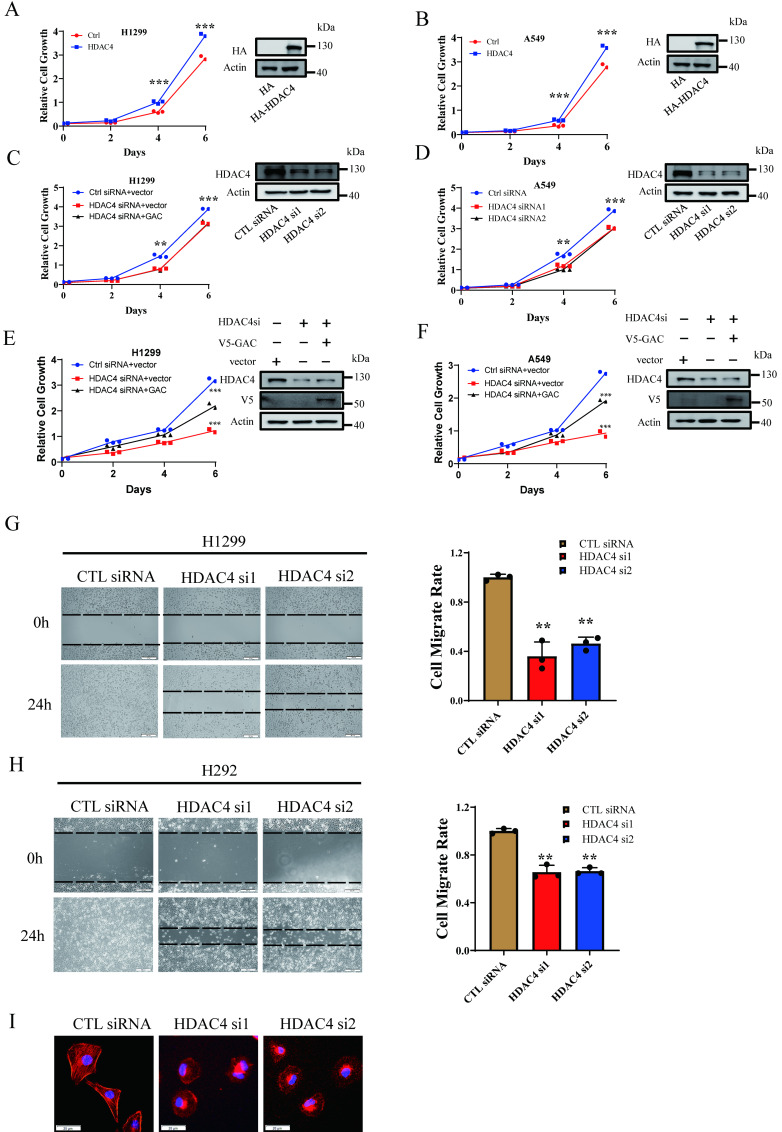
** HDAC4 promoted cell proliferation and migration in NSCLC cells. A, B** Indicated plasmids were transfected into H1299 (A) and A549 (B) cells and cell growth assay was performed. **C, D** Indicated siRNAs were transfected into H1299 (C) and A549 (D) cells and cell growth assay was performed. **E, F** Indicated siRNAs and plasmids were transfected into H1299 (E) and A549 (F) cells and cell growth assay was performed. Western blot assay was performed to confirm the transfection efficiency. **G, H** Indicated siRNAs were transfected into H1299 cells (G) and H292 cells (H) and cell wound healing assay was performed (scale bar: 500 µm, magnification: 100×). I F-actin staining assay. Indicated siRNAs were transfected into H1299 cells. 48 h later, cells were stained with phalloidin and DAPI. Scale bar=20 µm. Data are showed as mean ± SD, n=3. **P<0.01, ***P<0.001.

Our results above showed HDAC4 activated GAC, an oncogene involved in tumorigenesis and progression in various human cancers. Therefore, we investigated the effects of HDAC4 on cells proliferation and migration in NSCLC cells. HDAC4 overexpression promoted cell growth in H1299 and A549 cells (Fig. 3A, B). HDAC4 knockdown dramatically inhibited the proliferation of H1299 and A549 cells (Fig. 3C, D). Colony formation assay showed similar results. HDAC4 overexpression promoted colony formation while HDAC4 knockdown significantly decreased colony formation ability of NSCLC cells (Fig. S3A-D). To investigate if HDAC4 promoted cell growth through the regulation of GAC, we overexpressed V5-tagged GAC in HDAC4 knockdown cells. Interestingly, overexpression of GAC partially rescued the proliferative defect caused by HDAC4 knockdown in H1299 and A549 cells (Fig. 3E, F), suggesting that the biological function of HDAC4 is not restricted to the regulation of GAC.

Next, we evaluated the effect of HDAC4 on cell migration. Our results showed that cell migration was remarkedly inhibited in both H1299 and H292 cells when HDAC4 was knocked down by siRNAs (Fig. 3G, H). Statistical analysis indicated that the “wound” was healed in untreated H1299 and H292 cells after 24 h. On the contrary, the healing of “wound” was markedly reduced when HDAC4 was knocked down by siRNAs, and the migration rate was decreased to 40.6% in H1299 cells and 65.9% in H292 cells. Phalloidin staining assay showed that pseudopodia formation was greatly inhibited following HDAC4 knockdown in H1299 cells (Fig. 3I). The data above suggested HDAC4 played a vital role in NSCLC proliferation and migration.

### TRIM21 is the E3 ligase for GAC

**Figure 4:**
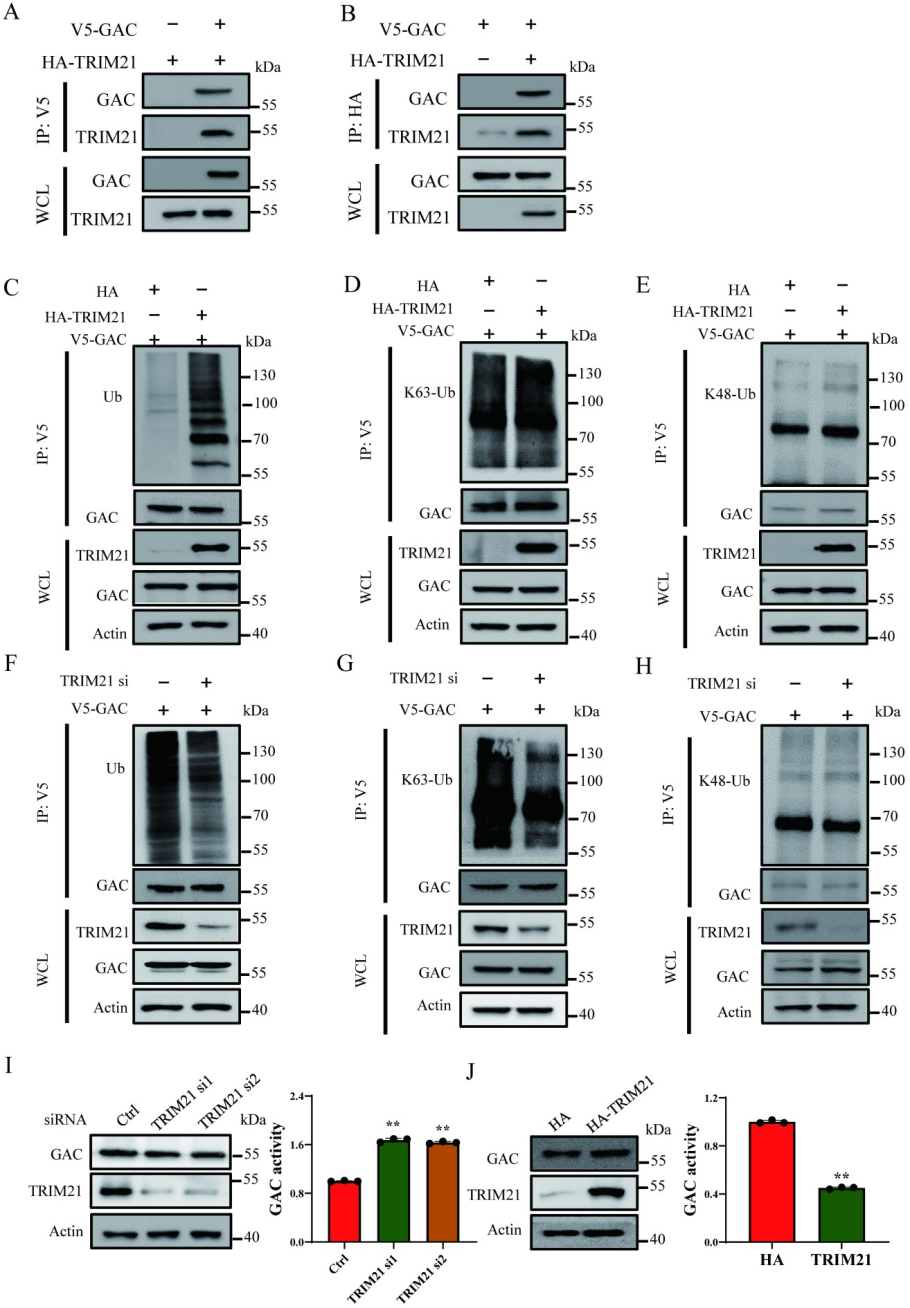
** TRIM21 is the E3 ligase for GAC. A, B** Indicated plasmids were transfected into H1299 cells. Interaction between GAC and TRIM21 were detected by immunoprecipitation and western blot assay. **C-E** Indicated plasmids were transfected into H1299 cells. The levels of GAC ubiquitination were detected by immunoprecipitation and western blot assay. **F-H** Indicated plasmids and siRNAs were transfected into H1299 cells. The levels of GAC ubiquitination were detected by immunoprecipitation and western blot assay. WCL: whole cell lysate. I Indicated siRNAs were transfected into H1299 cells. The protein expression was determined by western blot and glutaminase activity assay was performed. J Indicated plasmids were transfected into H1299 cells. The protein expression were determined by western blot and glutaminase activity assay was performed. Data are showed as mean ± SD, n=3. **P < 0.01.

Cross-talk between lysine ubiquitination and acetylation is an important regulatory mechanism in regulating protein functions. Interestingly, we observed that ubiquitination level of GAC markedly increased following NAM and TSA treatment (Fig. S4A), indicating a cross-talk between GAC acetylation and ubiquitination. Through co-overexpression of GAC and ubiquitin in H1299 cells, we found that GAC bound more to wild-type and K63-only ubiquitin than to K48-only ubiquitin (Fig. S4B). In order to elucidate the regulatory mechanism of GAC ubiquitination, we performed immunoprecipitation followed with mass spectrometry analysis, which identified proteins that interacted with GAC. There were two E3 ligase among the identified proteins in mass spectrometry analysis, TRIM21 (Tripartite motif containing-21) and XIAP (X-linked inhibitor of apoptosis) (Table S1). Immunoprecipitation and western blot results showed that TRIM21 indeed interacted with GAC in NSCLC cells but not XIAP (Fig. 4A, B; Fig. S5A, B). TRIM21 overexpression increased wild-type and K63-linked GAC ubiquitination in H1299 and A549 cells, while K48-linked GAC ubiquitination remained unchanged (Fig. 4C-E; Fig. S5C-E), suggesting that TRIM21 was the E3 ligase responsible for GAC ubiquitination. To further confirm our hypothesis, TRIM21was knocked down in H1299 and A549 cells. Our results showed that wild-type and K63-linked GAC ubiquitination were significantly decreased with no change in the K48-linked GAC ubiquitination (Fig. 4F-H and Fig. S5F-H). Next, we tested the effects of TRIM21 on GAC expression and activity, and found that TRIM21 knockdown in H1299 and A549 cells increased GAC activity (Fig. 4I; Fig. S5I). However, overexpression of TRIM21 in H1299 and A549 cells decreased GAC activity with no change in GAC expression (Fig. 4J; Fig. S5J). Taken together, we concluded that the interaction between TRIM21 and GAC led to K63-linked ubiquitination and inhibited GAC activity.

### Acetylation at Lys311 promotes GAC-TRIM21 interaction and GAC ubiquitination

**Figure 5:**
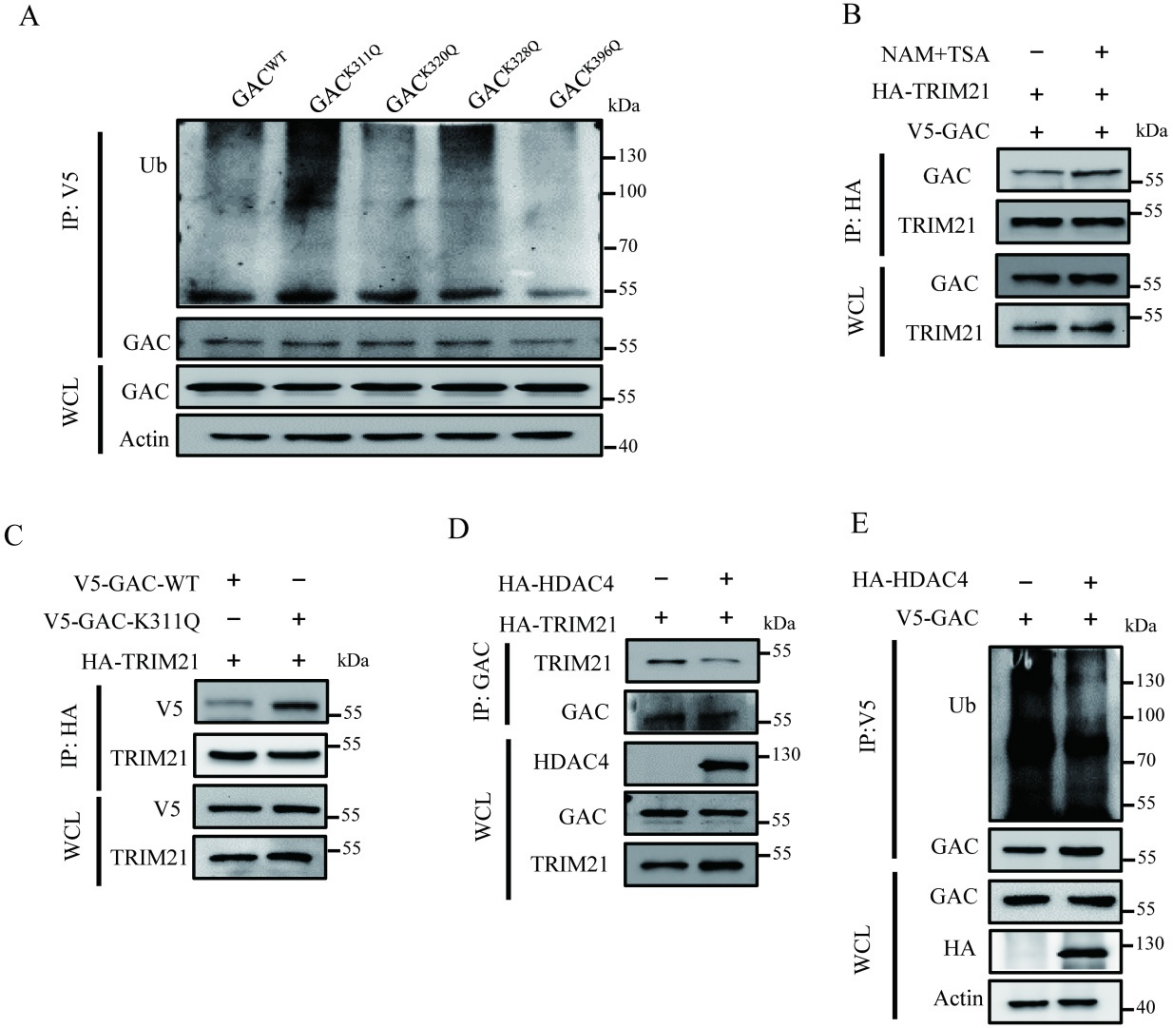
** Lys311 acetylation promotes GAC-TRIM21 interaction and GAC ubiquitination. A** Indicated plasmids were transfected into H1299 cells. Interaction between GAC and TRIM21 were detected by immunoprecipitation and western blot assay. **B** Indicated plasmids were transfected into H1299 cells followed by treatment with or without NAM and TSA. Interaction between GAC and TRIM21 were detected by immunoprecipitation and western blot assay. **C** Indicated plasmids were transfected into H1299 cells. Interaction between GAC and TRIM21 were detected by immunoprecipitation and western blot assay. **D** Indicated plasmids were transfected into H1299 cells. Interaction between GAC and TRIM21 were detected by immunoprecipitation and western blot assay. **E** Indicated plasmids were transfected into H1299 cells. The levels of GAC ubiquitination were detected by immunoprecipitation and western blot assay. WCL: whole cell lysate.

The interaction between E3 ligase and its substrates is crucial in regulation of protein ubiquitination. We investigated the role of Lys311 acetylation in TRIM21-dependent GAC ubiquitination. Our results showed that NAM and TSA treatment also increased GAC ubiquitination (Fig. S4A). This phenomenon was similar to that caused by acetylation-mimetic mutant K311Q (Fig. 5A; Fig. S6A). This crosstalk between GAC acetylation and ubiquitination prompted us to investigate the role of K311 acetylation in GAC ubiquitination. We then investigated the binding of GAC with TRIM21 in response to deacetylases inhibition. The binding of GAC to TRIM21 was readily detectable and was increased upon deacetylase inhibitor treatment in H1299 and A549 cells (Fig. 5B; Fig. S6B). Moreover, immunoprecipitation results showed that acetylation mimetic GAC-K311Q mutant bond to TRIM21 more than wild-type GAC (Fig. 5C; Fig. S6C). Since acetylation enhanced the interaction between GAC and TRIM21, we investigated the influence of HDAC4 overexpression on the association between GAC and TRIM21. Indeed, overexpression of HDAC4 in H1299 and A549 cells decreased interaction between GAC and TRIM21 (Fig. 5D; Fig. S6D), this result was further confirmed by the result that HDAC4 overexpression significantly decreased GAC ubiquitination in H1299 and A549 cells (Fig. 5E; Fig. S6E). All these results proved that Lys311 acetylation of GAC facilitated its binding to TRIM21.

### GAC acetylation inhibits tumorigenesis *in vivo*

**Figure 6:**
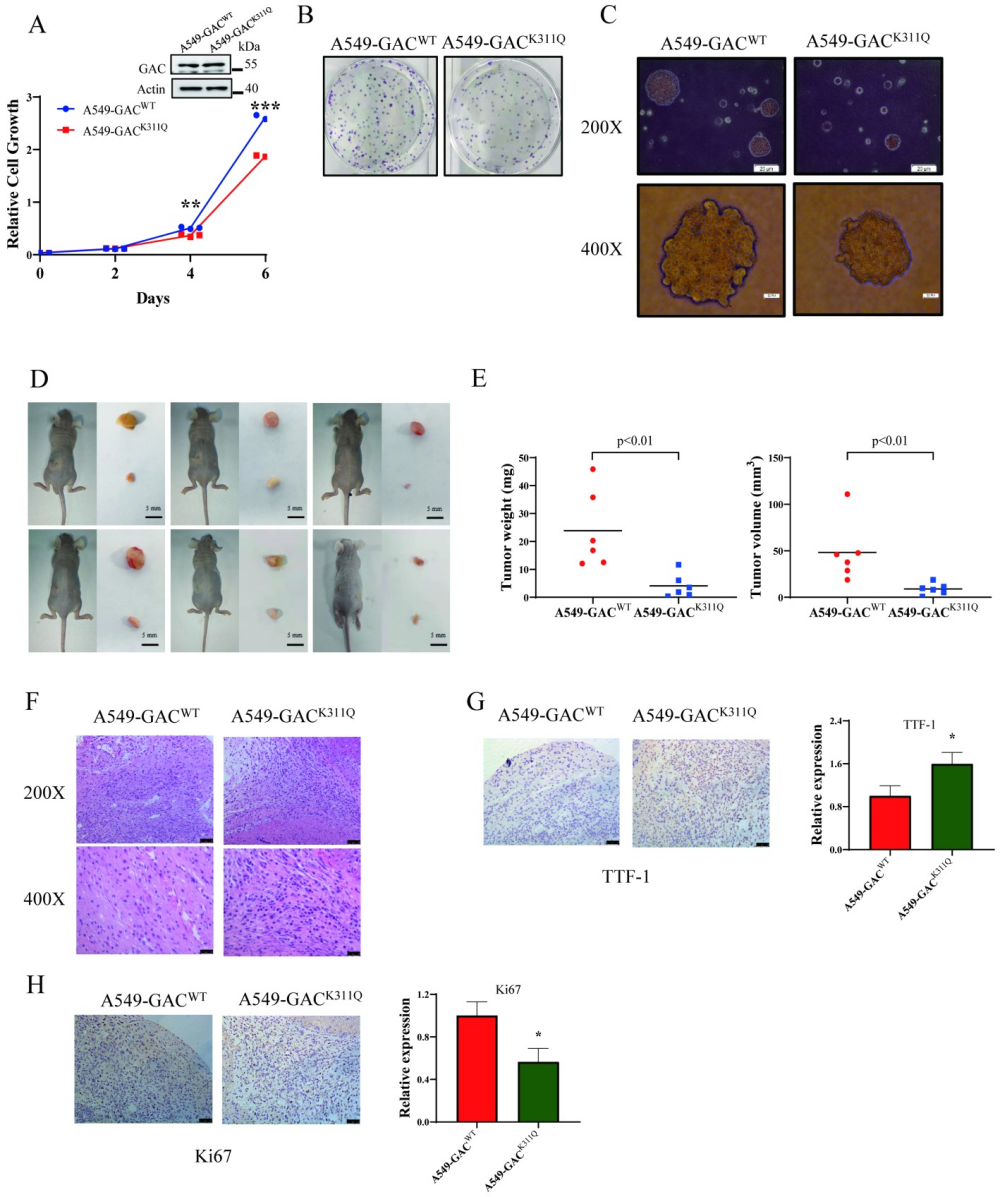
** GAC acetylation inhibits NSCLC progression *in vivo*. A** A549-GAC^WT^ and A549-GAC^K311Q^ stable cells were cultured for indicated times, then cell proliferation assay was performed. The expression of indicated proteins was determined by western blot with the indicated antibodies. **B** A549-GAC^WT^ and A549-GAC^K311Q^ stable cells were seeded in 6-well plates and colony formation assay was performed. **C** A549-GAC^WT^ and A549-GAC^K311Q^ stable cells were cultured in RPMI 1640 and soft agar assay was performed. Colonies were photographed after 14 days of growth. Scale bars of top figures is 20 µm. Scale bars of bottom figures is 50 µm. **D, E** Xenograft tumorigenesis. Nude mice were subcutaneously injected with parental A549-GAC^WT^ and A549-GAC^K311Q^ stable cells (1×10^7^). Tumors were dissected out and photographed (D) after four weeks, and then tumor weights and volumes were measured (E). **F** Tumors formed by parental A549-GAC^WT^ or A549-GAC^K311Q^ stable cells were subjected to hematoxylin-eosin (H&E) staining. Scale bars of top figures is 50 µm. Scale bars of bottom figures is 10 µm. **G, H** Immunohistochemical staining of tumors formed by A549-GAC^WT^ or A549-GAC^K311Q^ stable cells for Ki67 and TTF1. Scale bars of figures are 50 µm. Data are showed as mean ± SD, n=3. *P < 0.05, **P < 0.01, ***P < 0.001.

To investigate the influence of GAC acetylation on tumor proliferation *in vivo*, we screened out stable cell lines overexpressing GAC^WT^ or GAC^K311Q^, namely A549-GAC^WT^ and A549-GAC^K311Q^. We found that compared to A549 cells stably expressing wild-type GAC, A549 cells stably expressing GAC^K311Q^ showed slower cell proliferation rate (Fig. 6A), colony formation (Fig. 6B), and formed smaller colonies in soft agar assay (Fig. 6C). Then, we used xenograft assay to evaluate the tumorigenetic ability of A549-GAC^K311Q^ stable cells. Results showed that compared with A549-GAC^WT^ stable cells, tumors formed by A549-GAC^K311Q^ stable cells displayed reduced weight and size (Fig. 6D, E; Fig. S7). Hematoxylin-Eosin (H&E) staining results revealed that the tumors arose from the A549-GAC^WT^ stable cells had many larger cells and cell size was heterogeneous, indicating a very poor differentiation grade. However, tumors arose from A549-GAC^K311Q^ stable cells were homogeneous in cell size, suggesting a high differentiation grade (Fig. 6F). Next, we evaluated the expression of Ki67 and TTF-1 in tumors. Ki67 is a protein marker that is used in cell proliferation assessments 23. Our results showed that Ki67 expression was lower in tumors derived from A549-GAC^K311Q^ stable cells than in tumors formed by A549-GAC^WT^ stable cells (Fig. 6G). Thyroid transcription factor-1 (TTF-1) is a protein marker that is used in cell differentiation assessments 24. TTF-1 expression was higher in tumors derived from A549-GAC^K311Q^ stable cells than in tumors formed by parental A549-GAC^WT^ stable cells (Fig. 6H). These results indicated that GAC acetylation inhibited tumor progression in NSCLC.

### The overall effects of GAC acetylation on cell metabolism

**Figure 7:**
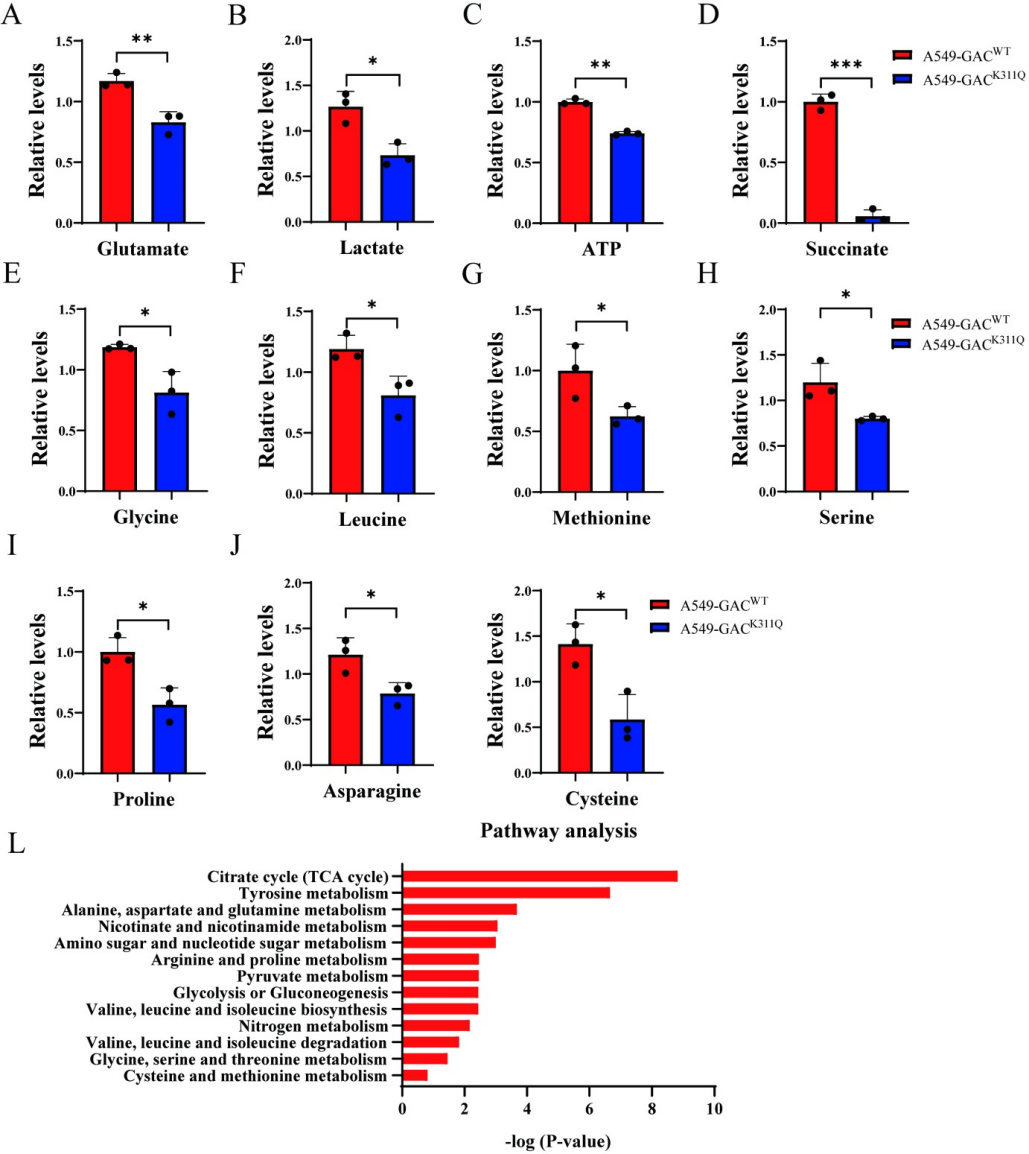
** The overall effects of GAC acetylation on cell metabolism and cancer-related pathways. A-K** Metabolomics analysis showed the relative levels of various metabolites in A549-GAC^WT^ and A549-GAC^K311Q^ stable cells. **L** Pathway analysis of decreased metabolites in A549-GAC^K311Q^ stable cells was shown. Data are showed as mean ± SD, n=3. *P < 0.05, **P < 0.01.

To figure out the metabolic changes associated with the different tumorigenetic ability, metabolomics analysis was performed to assay the concentrations of various metabolites in A549-GAC^WT^ cells and A549-GAC^K311Q^ cells. Compared with A549-GAC^WT^ stable cells, level of glutamate decreased significantly in A549-GAC^K311Q^ stable cells (Fig. 7A), indicating that high GAC acetylation decreased its activity and resulted in slow down glutaminolysis. The production of lactate and ATP were lower in A549-GAC^K311Q^ stable cells than in A549-GAC^WT^ stable cells, indicative of decreased glycolysis (Fig. 7B, C). Our results also showed that production of succinate in TCA cycle was lower in A549-GAC^K311Q^ stable cells than in A549-GAC^WT^ stable cells (Fig. 7D). Since TCA cycle is the center of cell metabolism, our finding indicated that the TCA cycle was also impaired when GAC activity was inhibited in A549-GAC^K311Q^ stable cells. Furthermore, the production of various amino acids decreased significantly in A549-GAC^K311Q^ stable cells comparing with A549-GAC^WT^ stable cells, indicating that the biosynthesis of amino acids was suppressed when the activity of GAC was inhibited (Fig. 7E-K). Pathway enrichment analysis of the decreased metabolites in the A549-GAC^K311Q^ stable cells included TCA cycle, amino acid metabolism and glycolysis (Fig. 7L). Taken together, these results indicated that GAC^K311Q^ mutation inhibited overall metabolism in lung cancer cells and explained the decreased tumorigenesis ability of A549-GAC^K311Q^ stable cells.

## Discussion

Metabolic reprogramming is a hall marker of tumorigenesis, in which amino acids play a crucial role in redox balance, homeostatic maintenance, biosynthetic support and energetic regulation 25. It has been reported that many amino acid metabolic enzymes, including glutaminase, were overexpressed or exhibited higher activity in tumor samples and cancer cells derived from patients 26, 27. The expression and activity of GAC, a major form of glutaminase in tumors, is upregulated in multiple cancers and contributes to tumorigenesis including in non-small cell lung cancer 10-13. Hence, it is important to illuminate the mechanism regarding GAC activity regulation in cancer cells.

Presently, we found that acetylation of GAC at Lys311 inhibited its activity and could be directly regulated by HDAC4. As a class II deacetylase, HDAC4 has been demonstrated to be implicated in various tumors. High HDAC4 expression promotes tumor progression in esophageal carcinoma and glioma and is associated with poor survival 28, 29. HDAC4 inhibition decreased cell proliferation, migration and metastasis in breast cancer, colorectal cancer as well as myeloma 30-32. Additionally, inhibition of HDAC4 sensitizes lung cancer to doxorubicin resistance and ionizing radiation 33, 34. However, the molecular mechanism regarding HDAC4 regulated tumor metabolism and tumorigenesis remains unclear until our current work. Our results showed that HDAC4 promotes the growth and migration of NSCLC cells, which is consistent with the findings in other cancers. We showed that HDAC4 directly interacted with GAC and deacetylated GAC at Lys311 in non-small cell lung cancer cells, which reduced GAC ubiquitination and increased GAC activity. Taken together, our findings revealed that HDAC4 promoted tumorigenesis by activating GAC and thus induced metabolic reprogramming to meet the biosynthetic and bioenergetic demands of cell growth and metastasis. On the other hand, GAC pan-acetylation increased following NAM treatment while NAM treatment had no effect on GAC Lys311 acetylation, suggesting that some acetylation sites other than Lys311 could be deacetylated by Sirtuin protein family, which might require further investigation. The supportive role of HDAC4 in cancer cell metabolic reprogramming, inhibition of HDAC4 sensitizing lung cancer to chemotherapy and radiotherapy 33, 34, all these phenomena support further investigation of HDAC4 as a potential therapeutic target.

Our study also showed that acetylation at Lys311 promoted the association between GAC and TRIM21 and increased K63-linked GAC ubiquitination. TRIM21 is an E3 ligase via its RING domain and is a member of the tripartite motif family 35. TRIM21 is related to innate immune response whose substrates include DDX41, IRF3, IRF5 and other interferon response factors 36-38. Recent studies showed that TRIM21 played a controversial role in tumor development. High expression level of TRIM21 indicated good prognosis in breast cancer, diffuse large B-cell lymphoma and hepatocellular carcinoma 39-41. However, TRIM21 overexpression promoted tumor progression by destabilizing p53 in glioma and increased cisplatin resistance in colon cancer cells by down-regulating Par-4 levels 42, 43. Our study identified GAC as a novel substrate of TRIM21 which regulated GAC activity in a Lys311-acetylation-dependent manner. Deacetylation of GAC at Lys311 reduced the interaction between GAC and TRIM21, resulting in GAC activation. Interestingly, by down-regulating TRIM21 and sequestering residual TRIM21 stress-fiber subset, NSCLC cells retained PFK expression and high glycolytic rates despite of environmental mechanics change 44. These results shed new light on TRIM21-targeted treatment in lung cancers.

The expression of Ki67 is strongly correlated to tumorigenesis and is widely used in routine pathological investigation as a reliable marker of proliferation and aggressiveness 45. The high expression level of Ki67 indicated a higher proliferation rate in tumors formed by a parental A549-GAC^WT^ stable cell line. On the contrary, tumors derived from parental A549-GAC^K311Q^ stable cells showed lower expression level of Ki67, indicating a low proliferation rate. TTF-1 expression is a diagnostic marker of cell differentiation 46. Tumors formed by A549-GAC^WT^ stable cells showed lower TTF-1 expression. In contrast, tumors derived from A549-GAC^K311Q^ stable cells showed higher expression level of TTF-1. Our findings indicated that inhibiting glutamine metabolism by GAC acetylation led low-differentiated lung adenocarcinomas to high-differentiated tumors.

In order to further elucidate the mechanism regarding different differentiation rate of tumors formed by A549-GAC^WT^ and A549-GAC^K311Q^ stable cells, we performed metabolomic analysis. Our results showed impaired glutamine metabolism in A549-GAC^K311Q^ cells, as indicated by reduced production of glutamate. Production of lactate and succinate also decreased in A549-GAC^K311Q^ stable cells, suggesting that glycolysis and citric acid cycle were downregulated. Pathway enrichment analysis also showed that several amino acid metabolism pathways were downregulated following GAC^K311Q^ mutation. These results demonstrated that inhibiting glutamine metabolism led to metabolic reprogramming which contributed to reduced proliferation rate and high differentiation level in NSCLC cells.

**Figure 8:**
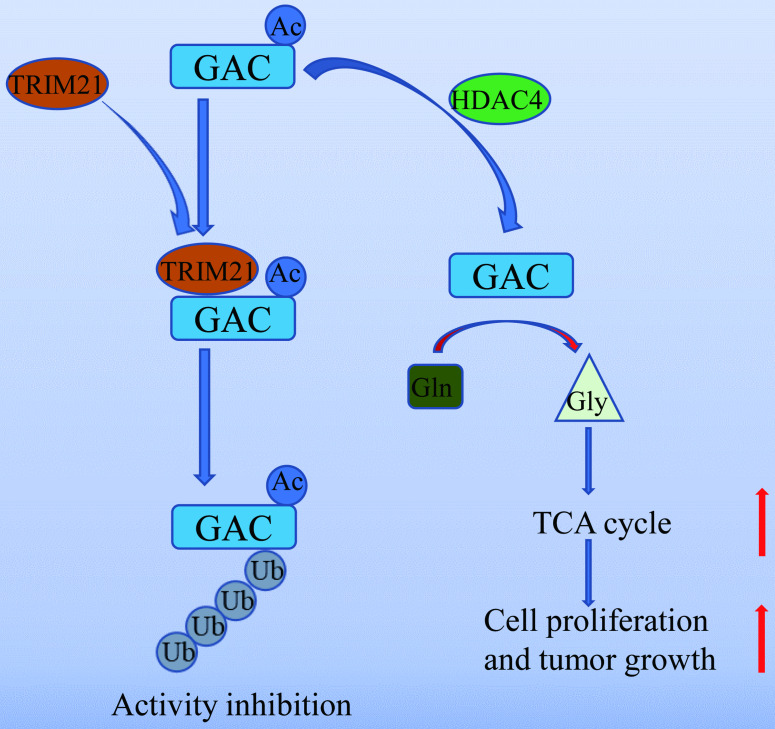
** A working model of GAC acetylation in NSCLC.** A working model depicting the molecular mechanism of HDAC4 mediated GAC deacetylation and TRIM21 mediated GAC ubiquitination to regulation tumorigenesis in NSCLC.

In summary, our work unveiled a novel mechanism of GAC regulation by acetylation and ubiquitination which participated in NSCLC tumorigenesis (Fig. 8). Our study further supported the notion that GAC could be a valuable therapeutic target for NSCLC.

## Supplementary Material

Supplementary figures and table.

## Abbreviations

HIF: Hypoxia-inducible factor
lncRNA: long non-coding RNA
NAM: nicotinamide
NSCLC: non-small cell lung cancer
PTM: post-translational modifications
TRIM21: Tripartite motif containing-21
TSA: trichostatin A
TTF-1: Thyroid transcription factor-1
XIAP: X-linked inhibitor of apoptosis
