# Supplementary material for: Deacetylation of Glutaminase by HDAC4 contributes to Lung Cancer Tumorigenesis
Source: Int J Biol Sci. 2022 Jul 4;18(11):4452–65. doi: 10.7150/ijbs.69882 (PMC9295053; doi:10.7150/ijbs.69882)
Supplement: Supplementary file 1 — Supplementary figures and table. [file ijbsv18p4452s1.pdf]

Table. S1 Identification of GAC interacting E3 ligase.

Reference	Score	Area	MV
GAC	156.81	2.271E9	61.0
XIAP	10.23	8.901E6	56.6
TRIM21	6.27	1.043E7	54.1

Table. S1 Identification of GAC interacting E3 ligase. GAC interacting proteins involved in ubiquitination were shown.

Figure S1

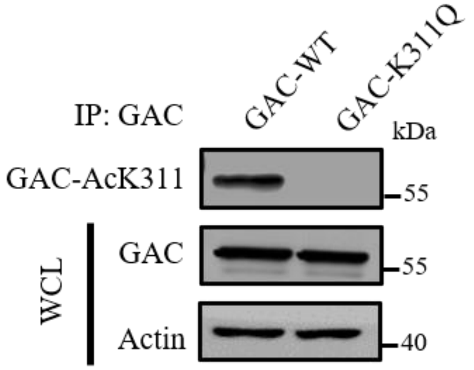

Fig. S1 Verification of the specificity of GAC-K311Ac antibody. Indicated plasmids were transfected into H1299 cells. GAC acetylation was detected by immunoprecipitation and western blot assay were performed. WCL: whole cell lysate.

Figure S2

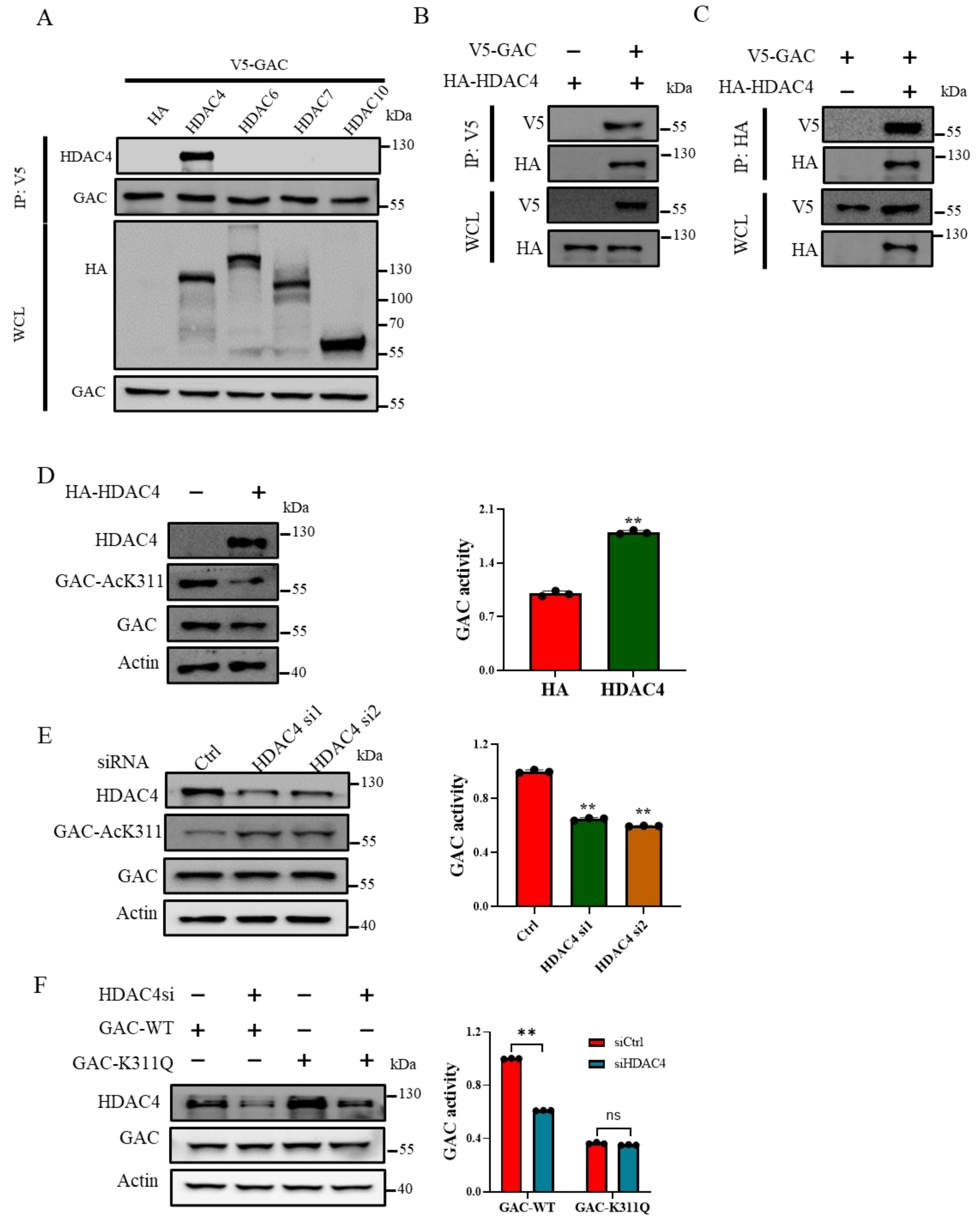

A
V5-GAC
HA
HDAC4
HDAC6
HDAC7
HDAC10
kDa
IP: V5
HDAC4
GAC
WCL
HA
GAC
130
55
130
100
70
55
55
B
V5-GAC
HA-HDAC4
kDa
IP: V5
V5
HA
WCL
V5
HA
55
130
55
130
C
V5-GAC
HA-HDAC4
kDa
IP: HA
V5
HA
WCL
V5
HA
55
130
55
130
D
HA-HDAC4
kDa
HDAC4
GAC-AcK311
GAC
Actin
130
55
55
40
GAC activity
2.1
1.4
0.7
0.0
**
HA
HDAC4
E
siRNA
Ctrl
HDAC4 si1
HDAC4 si2
kDa
HDAC4
GAC-AcK311
GAC
Actin
130
55
55
40
GAC activity
1.2
0.8
0.4
0.0
**
**
Ctrl
HDAC4 si1
HDAC4 si2
F
HDAC4si
GAC-WT
GAC-K311Q
kDa
HDAC4
GAC
Actin
130
55
40
GAC activity
1.2
0.8
0.4
0.0
**
ns
siCtrl
siHDAC4
GAC-WT
GAC-K311Q

Fig. S2 HDAC4 is responsible for deacetylation of GAC at K311. A Indicated plasmids were transfected into 293T cells. Interaction between HDACs and GAC was detected by immunoprecipitation and western blot. B, C Indicated plasmids were transfected into A549 cells. Interaction between HDAC4 and GAC was detected by immunoprecipitation and western blot. WCL: whole cell lysate.D Indicated plasmids were transfected into A549 cells. The protein expressions were determined by western blot and glutaminase activity assay was performed. E Indicated siRNAs were transfected into A549 cells. The protein expressions were determined by western blot and glutaminase activity assay was performed. F Indicated plasmids and siRNAs were transfected into A549 cells. Cell lysates were immunoprecipitated with anti-V5 antibody and then glutaminase activity assay was performed. Data are showed as mean ± SD, n=3. **$P < 0.01$, ns $P > 0.05$.

Figure S3

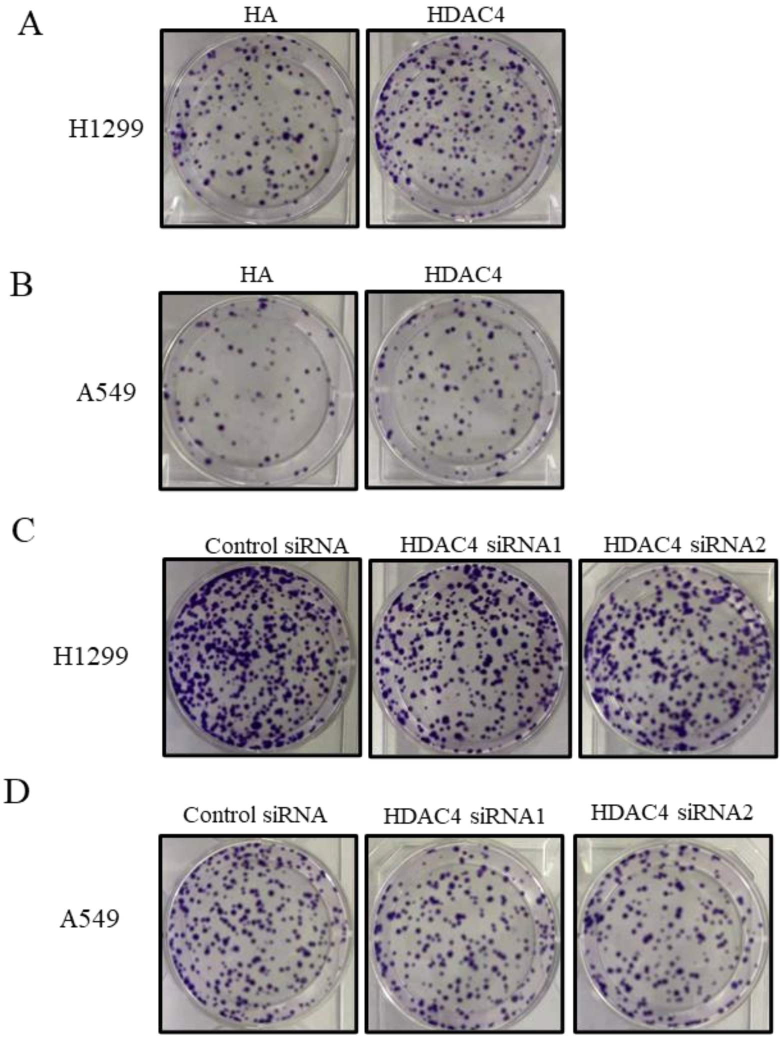

Fig. S3 HDAC4 promotes cell proliferation in NSCLC. A-D Indicated plasmids or siRNAs were transfected into NSCLC cells (H1299 and A549). 24h later, Cells were counted and seeded in 6-well plates and colony formation assay was performed.

Figure S4

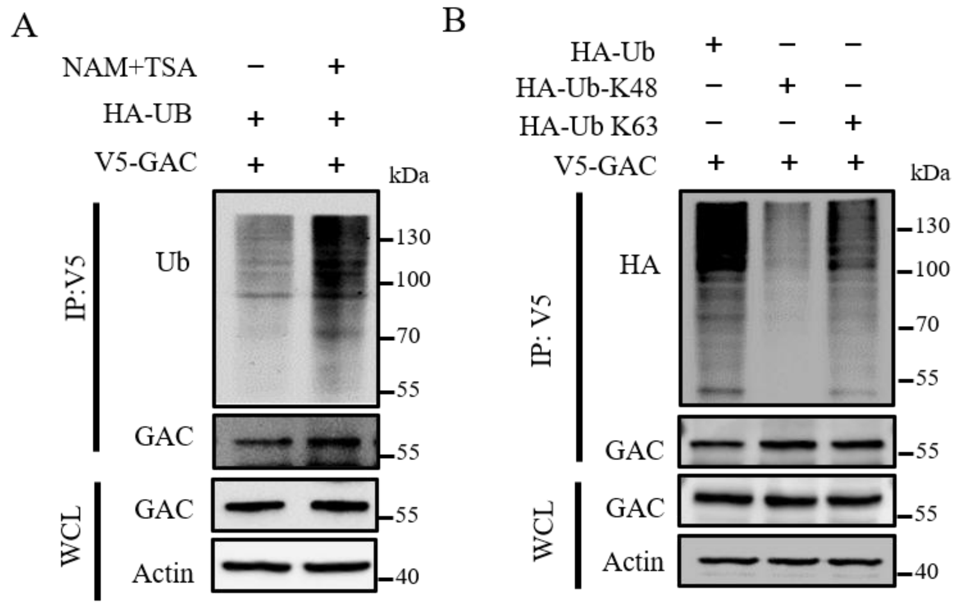

Fig. S4 Ubiquitination of GAC. A Indicated plasmids were transfected into H1299 cells followed by treatment with or without NAM and TSA. The levels of GAC ubiquitination were detected by immunoprecipitation and western blot assay. B Indicated plasmids were transfected into H1299 cells. The type of GAC ubiquitination was detected by immunoprecipitation and western blot assay. WCL: whole cell lysate.

Figure S5

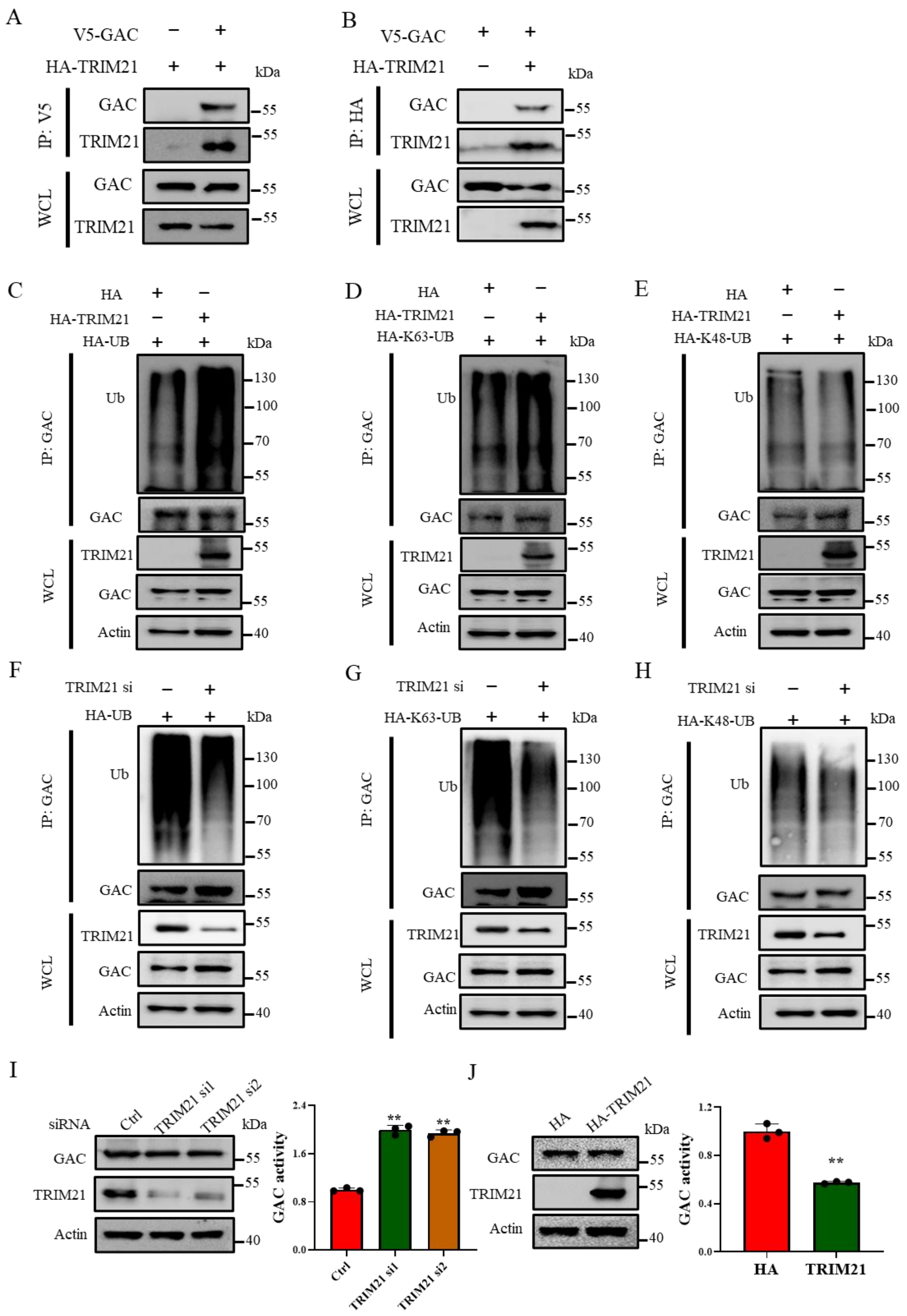
A
V5-GAC
HA-TRIM21
kDa
IP: V5
WCL
GAC
TRIM21
B
IP: HA
C
HA
HA-UB
IP: GAC
Ub
Actin
D
HA-K63-UB
E
HA-K48-UB
F
TRIM21 si
G
H
I
siRNA
Ctrl
TRIM21 si1
TRIM21 si2
GAC activity
J
HA-TRIM21
TRIM21

Supplementary Fig. S5 TRIM21 is the E3 ligase for GAC. A, B Indicated plasmids were transfected into A549 cells. Interaction between TRIM21 and GAC was detected by immunoprecipitation and western blot. C-E Indicated plasmids were transfected into A549 cells. The levels of GAC ubiquitination were detected by immunoprecipitation and western blot assay. F-H Indicated plasmids and siRNAs were transfected into A549 cells. The levels of GAC ubiquitination were detected by immunoprecipitation and western blot assay. WCL: whole cell lysate. I Indicated siRNAs were transfected into A549 cells. The protein expressions were determined by western blot and glutaminase activity assay was performed. J Indicated plasmids were transfected into A549 cells. The protein expressions were determined by western blot and glutaminase activity assay was performed. Data are showed as mean $\pm$ SD, n=3. ** $P < 0.01$.

Figure S6

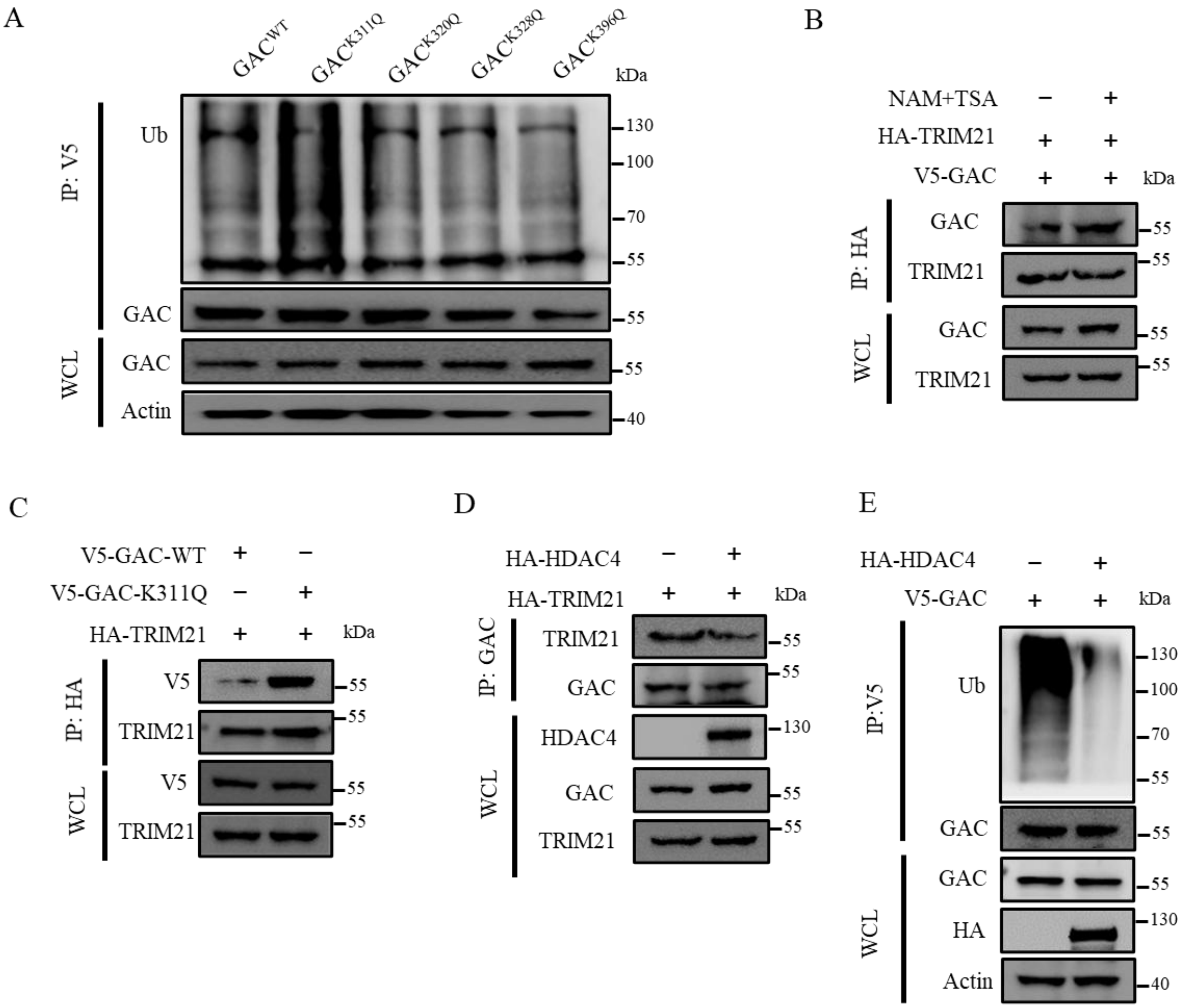

Fig. S6 Lys311 acetylation promotes GAC-TRIM21 interaction and GAC ubiquitination. A Indicated plasmids were transfected into A549 cells. Interaction between TRIM21 and GAC was detected by immunoprecipitation and western blot. B Indicated plasmids were transfected into A549 cells followed by treatment with or without NAM and TSA. Interaction between TRIM21 and GAC was detected by immunoprecipitation and western blot. C Indicated plasmids were transfected into A549 cells. Interaction between TRIM21 and GAC was detected by immunoprecipitation and western blot. D Indicated plasmids were transfected into A549 cells

Interaction between TRIM21 and GAC was detected by immunoprecipitation and western blot. E Indicated plasmids were transfected into A549 cells. The levels of GAC ubiquitination were detected by immunoprecipitation and western blot assay. WCL: whole cell lysate.

Figure S7

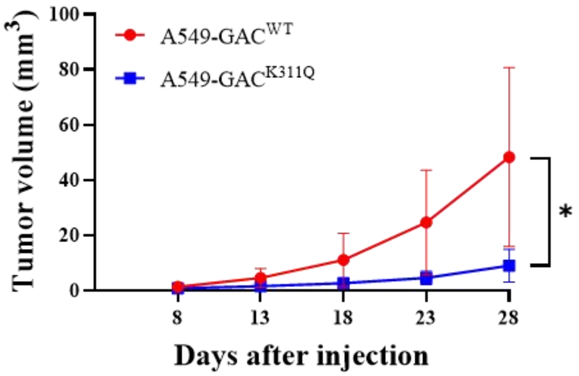

Fig. S7 Time course changes of tumor volumes. Nude mice were subcutaneously injected with parental A549-GAC^{WT} and A549-GAC^{K311Q} stable cells (1×10^7). Tumor volumes was measured starting from 8 days after injection. Data are showed as mean ± SD, n=6. * P< 0.05.
